# Genome-wide association and identification of candidate genes for age at puberty in swine

**DOI:** 10.1186/s12863-016-0352-y

**Published:** 2016-02-29

**Authors:** Dan J. Nonneman, James F. Schneider, Clay A. Lents, Ralph T. Wiedmann, Jeffrey L. Vallet, Gary A. Rohrer

**Affiliations:** United States Department of Agriculture, Agricultural Research Service, U.S. Meat Animal Research Center, Clay Center, NE 68933 USA

**Keywords:** Bayes, Genome-wide association, Puberty, Pig, SNP, Swine

## Abstract

**Background:**

Reproductive efficiency has a great impact on the economic success of pork production. Gilts comprise a significant portion of breeding females and gilts that reach puberty earlier tend to stay in the herd longer and be more productive. About 10 to 30 % of gilts never farrow a litter and the most common reasons for removal are anestrus and failure to conceive. Puberty in pigs is usually defined as the female’s first estrus in the presence of boar stimulation. Genetic markers associated with age at puberty will allow for selection on age at puberty and traits correlated with sow lifetime productivity.

**Results:**

Gilts (*n* = 759) with estrus detection measurements ranging from 140–240 days were genotyped using the Illumina PorcineSNP60 BeadChip and SNP were tested for significant effects with a Bayesian approach using GenSel software. Of the available 8111 five-marker windows, 27 were found to be statistically significant with a comparison-wise error of *P* < 0.01. Ten QTL were highly significant at *P* < 0.005 level. Two QTL, one on SSC12 at 15 Mb and the other on SSC7 at 75 Mb, explained 16.87 % of the total genetic variance. The most compelling candidate genes in these two regions included the growth hormone gene (*GH1*) on SSC12 and *PRKD1* on SSC7. Several loci confirmed associations previously identified for age at puberty in the pig and loci for age at menarche in humans.

**Conclusions:**

Several of the loci identified in this study have a physiological role for the onset of puberty and a genetic basis for sexual maturation in humans. Understanding the genes involved in regulation of the onset of puberty would allow for the improvement of reproductive efficiency in swine. Because age at puberty is a predictive factor for sow longevity and lifetime productivity, but not routinely measured or selected for in commercial herds, it would be beneficial to be able to use genomic or marker-assisted selection to improve these traits.

**Electronic supplementary material:**

The online version of this article (doi:10.1186/s12863-016-0352-y) contains supplementary material, which is available to authorized users.

## Background

Puberty is the process of physical maturation of an animal to be capable of sexual reproduction. In humans puberty may be defined as age at first menses in girls, a milestone well-recalled and widely recorded. In pigs puberty is defined as age at first estrus when an animal will stand for breeding. With these definitions, puberty manifests as a spontaneous event. In reality, attainment of puberty is a complex maturation process that involves multiple body tissues and organ systems [1–3]. The hypothalamus receives neural and endocrine input from these systems to appropriately activate the pituitary-ovarian axis under conditions favorable for successful pregnancy to occur. Timing of puberty varies widely within and between populations and can be associated with several adult conditions and phenotypes. Increased risk for adult ovarian cancer [4–6], endometrial cancer [7, 8] and obesity [9–11] have been associated with early puberty in girls. Girls that reach puberty later have lower fertility [12, 13]. Similarly in pigs, later age at puberty is associated with lower fertility. Gilts that have an earlier age at first estrus stay in the herd longer, are more likely to farrow multiple litters, give birth to more piglets and thus have a longer more productive life [14, 15].

Gilts comprise a significant portion of breeding females and thus successful gilt development is critical to overall herd performance. Management decisions prior to first mating of gilts can affect productivity and later reproductive performance [16, 17]; however, estrous traits of gilts (e.g., duration of estrus) are genetically correlated to adult reproductive phenotypes such as wean to estrus interval and sow longevity [18], making age at first estrus an early indicator trait that can be used to select for favorable adult reproductive performance. Age at puberty is moderately heritable in pigs (h^2^ = 0.38 to 0.46) [19, 20]; but, age at first estrus is rarely recorded in pork production due to limitations on costs, labor, and time. To facilitate genetic change in livestock, traits that are typically not recorded, sex-limited, or only measurable in adulthood, but that are critical for important selection decisions early in life, are ideal candidates to consider for implementing whole-genome selection [21]. A significant number of genomic loci associated with age at menarche have been identified in humans through GWAS [22–24]. With the development of a commercially available, high-density SNP array for pig [25], genome-wide selection and the identification of genes and pathways affecting age at puberty of swine is feasible. Genome-wide associations for age at puberty [26] and delayed puberty [27] in the pig have previously been described. Several of the candidate genes associated with age at puberty are expressed in tissues from the hypothalamic-pituitary-ovarian axis [26] and are involved in sexual and social behavior, energy balance and oocyte maturation. Candidate genes associated with delayed puberty are similarly expressed but are more involved with synchronization of reproductive behavior and ovulation [27]. The objectives of this study were to use high-density genotyping and genome-wide association analysis to identify chromosomal regions and genes influencing age at puberty in a line of white-composite pigs and determine the proportion of genetic variance explained by the markers.

## Methods

Care and handling of all animals included in this study was according to procedures outlined in *Guide for Care and Use of Agricultural Animals in Agricultural Research and Teaching* [28] and approved by the USMARC Animal Care and Use Committee.

### Animals and data

A composite population was developed in 2001 using maternal and terminal Landrace, Duroc, and Yorkshire lines. Full- and half-sib matings were prevented, otherwise matings were random. Twelve original sire-lines were maintained and semen from all sire-lines was used to produce approximately 600 litters per generation. Additional details of the development of this population were previously reported [29]. Gilts born during 2005 through 2008 (*n* = 759) were genotyped and used for this study.

Estrus detection was performed daily from 140–240 days using 5–6 mature boars (>11 mo of age) placed in an alleyway between two pens of gilts, during which time herdsmen applied back pressure to gilts within each pen and observed them for estrous behavior. Age at puberty was defined as the date in which the first standing estrus was detected. Gilts that did not show signs of estrus by 240 days were harvested at the USMARC abattoir at an average age of 241 days, and the ovaries were inspected to determine whether they had not cycled and were classified as nonpubertal, or had cycled and were classified as behaviorally anestrus. Gilts that were observed to reach puberty before slaughter age had an average age of 200 ± 14.5 (mean ± SD) days at first estrus.

### DNA isolation, SNP array genotyping and quality control

Genomic DNA was extracted from frozen tail tissue using the Wizard SV Genomic DNA Purification kit (Promega, Madison, WI, USA) for all phenotyped pigs. Samples of 300 ng at a concentration ≥ 75 ng/μl of DNA were genotyped using the Illumina PorcineSNP60 BeadChip containing 64,232 SNP (Illumina, San Diego, CA,USA) [25]. Genotypic reactions were completed at U.S. Meat Animal Research Center (USMARC, Clay Center, NE, USA) and then scanned at the USDA, ARS, Bovine Functional Genomics Laboratory (Beltsville, MD, USA). Scan results were interpreted at USMARC using Illumina’s BeadStudio Genotyping software. Genotypes were called for 59,895 SNP spanning the entire porcine genome. Chromosome and position locations for each marker were according to the *Sus scrofa* genome assembly 10.2 (http://www.ncbi.nlm.nih.gov/projects/mapview/map_search.cgi?taxid=9823).

Any SNP with unknown chromosome positions, those located on SSCY, those with call rates < 95 %, or minor allele frequencies < 0.05 were excluded from the data set. Animals were eliminated (*n* = 7) if > 5 % of SNP were missing or for failing a Mendelian segregation (parentage) test. After utilizing these quality control measures, a total of 41,148 SNP out of a total of 64,232 SNP on the array qualified for GWAS. A genotypic principal components analysis was done using genotypes of all 759 phenotyped animals with SNP & Variation Suite v8.4.1 (Golden Helix, Inc., Bozeman, MT, http://goldenhelix.com/) [30]. The first three principal components are plotted in Additional file 1: Figure S1 and does not show population stratification due to breed.

### Genome-wide association analyses

The analyses were implemented with a Bayes C model averaging approach using version 4.61 GENSEL software (http://archive.is/bigs.ansci.iastate.edu). Bayes C uses a common SNP variance that is reliably estimated from the data. The Bayes C has been explained previously by Kizilkaya et al. [31].

The following modified statistical model from Kizilkaya et al. [31] was used:

$$ \mathbf{y} = \mathbf{X}\;\boldsymbol{\beta} +\boldsymbol{Z}\boldsymbol{u}+\boldsymbol{e} $$

where **y** is the vector of phenotypes (age at puberty), **X** is an incidence matrix of fixed effects (***β***), **Z** is a matrix of SNP genotypes that were fitted as random effects (***u***) distributed N (**0, σ**^**2**^_**u**_), and **e** is the vector of random residual effects assumed to be normally distributed N (**0, σ**^**2**^_**e**_). A fixed classification factor used in this statistical model was year-season of birth as all females born in the same season were uniformly managed and monitored for first estrus.

Priors for variance components were taken from residual and additive genetic variance components from a preliminary analysis using MTDFREML [32]. The model fitted was:

$$ \mathbf{y}=\mathbf{X}\mathbf{b}+\mathbf{Z}\mathbf{a}+\mathbf{e} $$

where **y** represented a vector of observations; **b** was a vector of fixed effects; **a** was a vector of random additive genetic effects of animals, which was assumed to be distributed N (**0, A**σ_a_^2^), where **A** was the numerator relationship matrix among animals and **e** was a vector of residual effects, which was assumed to be distributed N (**0, I**σ_e_^2^) and where **I** was the identity matrix. Incidence matrix **X** related records to fixed effects and incidence matrix **Z** related records to additive genetic random effects. The fixed effects were those used in the Bayesian model above. The four generation pedigree file included 37 sires, 380 dams and 759 females with phenotypic data.

Bayes Cπ option of GENSEL was used to estimate $\boldsymbol{\uppi}$ at 0.9935 where $\boldsymbol{\uppi}$ is the prior probability that any SNP would have a zero effect. Bayes C was utilized for the analysis of SNP effects with a burn-in of 1000 iterations and a total of 51,000 iterations in a Markov chain. The results from this analysis included posterior distributions for the effects of each of the 41,148 markers, adjusted for the portfolio of all the other fitted marker effects in the model.

After the Bayes C analysis, the Predict option of GENSEL was used to estimate the genetic variance of sliding windows of five consecutive SNP assigned by chromosome-position order beginning with the first five SNP on SSC1 and ending with the last five SNP on SSCX. There were 8111 non-overlapping five-SNP windows available in the whole pig genome for statistical testing. Based on our estimate of π, 267 SNP effects were expected to be important (41,148 x (1-π) = 267). Therefore we submitted 267 5-SNP windows explaining the greatest proportion of genetic variance, defined as putative QTL, for statistical testing utilizing the Bootstrap option of GENSEL.

### Bootstrap analysis for hypothesis testing

To construct the distribution of the test statistic (genetic variance of a five-SNP window) for each putative QTL, bootstrap samples were produced using the posterior means of the 41,148 SNP. This involved creating 1000 bootstrap data sets [33–35]. These bootstrap samples were constructed according to the null hypothesis of no QTL in the identified SNP window. Construction of the bootstrap begins with the results of the Bayes C analysis including the SNP effects of all markers except the SNP that are within the region of the putative QTL, which are set to zero, and the estimates of the fixed effects relevant to each animal’s phenotypic record. Residual effects are sampled according to the residual variance previously found and added to each record. The only difference between bootstrap data replicates was due to different residual effects being sampled in different replicates. Each bootstrap sample was reanalyzed using the same Bayes C model used for the real data, and the genetic variances of the SNP window corresponding to the QTL were accumulated for comparison with the test statistic represented by the genetic variance of the SNP window identified in the analysis of the real data. If just one bootstrap statistic from the 1000 simulated exceeded the test statistic from the real data, the comparison-wise *P*-value was determined to be 0.001 < *P* < 0.002.

Multiple testing was taken into account considering the proportion of false positives [36]. This approach controls the proportion of false positive conclusions across all tests undertaken, rather than the probability of making one mistake over all tests, as would be the interpretation of an experiment-wise error correction. The proportion of false positives is calculated as a function of the average comparison-wise Type I error rate, the proportion of true null hypotheses tested among all hypotheses tested, and the power of the test.

### Candidate gene search

Intervals for candidate gene searches were defined as the 5-SNP window plus the 300 kb flanking regions in QTL peaks. Annotated genes contained in this window were identified using Ensembl BioMart tool (http://ensembl.org/Sus_scrofa/Info/Index) and the UCSC Genome Browser (http://genome.ucsc.edu/cgi-bin/hgGateway) with the Sus scrofa Build 10.2 assembly.

### Linkage disequilibrium (LD) analysis

Linkage disequilibrium (r^2^) was estimated for the SNP using Haploview 4.0 software [37] (http://www.broad.mit.edu/mpg/haploview/index.php). Haplotype blocks were based on pairwise LD values.

## Results and discussion

In the present study, a GWAS using the PorcineSNP60 BeadChip was performed by means of Bayes C model averaging with random SNP effects for age at puberty. The variances, heritability, and the proportion of total variance explained by the markers are shown in Table 1. Using MTDFREML, heritability was found to be moderate at 0.319, similar to heritabilities of 0.38–0.46 that have been reported before [19, 20]. Rothschild and Bidanel [38] summarized age at puberty heritability estimates and determined a mean of 0.33 with a range of 0.00 to 0.64. The proportion of total variance explained by the markers was estimated by GENSEL to be 0.11 and indicates that the SNP explain less genetic variation than the infinitesimal model. While both measures of heritability should have similar expectations, the reason for variation between the two methods in unknown. In our previous studies on litter traits, similar variability was seen between pedigree-based and marker-based estimates of heritability [29]. Additional file 2: Table S1 presents information about the 222 5-SNP window associations identified in this study including percent of genetic variance (GV) explained by each QTL, and bootstrap *P*-values. Twenty seven QTL regions were significantly (*P* < 0.01) associated with age at puberty after bootstrap analysis (Table 2). Fifteen of these were the highest ranked for genetic variance. Statistical testing identified one QTL with *P* < 0.001, nine QTL with 0.001 < *P* < 0.005, and 17 QTL with 0.005 < *P* ≤ 0.010. The QTL were identified on all autosomes as well as SSCX and explained 29.4 % of the GV identified by SNP markers tested. Sixteen additional putative QTL with *P* ≤ 0.027 located in the vicinity of a significant QTL are also listed in Additional file 2: Table S1.

**Table 1:** Summary statistics for age at puberty in pigs

Method	Number of Animals	Marker Heritability^1^	Genetic Variance	Residual Variance	Total Variance
MTDFREML	752	0.319 ± 0.102^2^	63.0	134.8	197.8
GenSel Bayes C	752	0.111	20.9	167.0	187.9

^1^Marker heritability (h^2^) is the proportion of total variance explained by the markers

^2^Only the standard error for heritability was provided by MTDFREML and no standard errors were provided by GenSel

**Table 2:** Most significant QTL for age at puberty in pigs

SSC	Start^1^	End^1^	Marker 1	Marker 5	GenVar (%)	Rank^2^	p-value	-log(p)	Gene	Human trait or function
12	14,910,939	15,006,310	M1GA0016274	ASGA0053410	9.7332	1	0.0001	4.000	*GH1*	growth/height
6	68,823,187	68,890,361	ASGA0091283	ALGA0035583	0.3064	35	0.001	3.000	*TMEM51*	
1	291,202,322	291,277,150	ALGA0009830	M1GA0001507	0.6121	10	0.003	2.523	*BRINP1*	
3	26,517,667	26,631,496	MARC0007734	ALGA0018104	0.5091	15	0.003	2.523	*GPR139*	
7	43,190,587	43,400,560	ALGA0040857	ASGA0033098	0.1673	75	0.003	2.523	*UBR2*	obesity
7	75,121,635	75,460,294	INRA0026429	H3GA0022045	7.1362	2	0.003	2.523	*C14orf23, PRKD1*	BMI/obesity
15	42,527,231	43,052,985	H3GA0044224	ALGA0114567	1.7467	3	0.003	2.523	*ANGPT2*	ovulation
3	11,143,547	11,286,978	H3GA0008684	ASGA0013430	0.5400	13	0.004	2.398	*GTF2IRD1*	
9	142,232,369	142,338,853	ASGA0044888	ASGA0044901	1.4283	4	0.004	2.398	*SMYD2*	
10	18,347,386	18,665,600	ASGA0046792	DRGA0010326	0.3992	24	0.004	2.398	*SDCCAG8*	obesity
3	11,960,567	12,093,180	ALGA0017578	ALGA0017611	1.0870	6	0.005	2.301	*GATSL2*	
14	138,428,984	138,850,067	DRGA0014684	ALGA0082115	0.4572	19	0.005	2.301	*SLC18A2*	dopamine transport
1	121,626,075	121,978,687	M1GA0001099	ASGA0004239	0.4196	21	0.006	2.222	*RORA*	menarche
3	21,596,232	22,794,763	ASGA0013855	ASGA0094123	0.1765	70	0.006	2.222	*AQP8*	menarche
3	27,054,931	27,208,960	MARC0085816	ALGA0124353	1.1368	5	0.006	2.222	*GPRC5B*	BMI/obesity
6	127,194,648	127,655,907	MARC0001714	ASGA0029572	0.3900	25	0.006	2.222	*ACADM*	metabolite levels
15	148,542,045	148,722,557	ASGA0071543	ASGA0071569	0.1564	83	0.007	2.155	*HJURP*	
8	15,600,021	15,671,195	DRGA0008334	ASGA0037920	0.2543	41	0.008	2.097	*SLIT2*	
8	109,067,052	109,213,652	H3GA0025237	MARC0017963	0.2658	39	0.008	2.097	*TRPC3*	
11	55,668,610	55,844,999	ALGA0062350	ALGA0062355	0.3136	34	0.008	2.097	*RNF219*	
16	44,362,264	44,481,215	ALGA0090494	MARC0073104	0.2123	50	0.008	2.097	*IPO11*	
18	50,837,228	51,030,054	DRGA0017050	ASGA0105879	0.5142	14	0.008	2.097	*SNX10*	visceral adipose tissue
2	133,313,068	133,376,955	INRA0009763	MARC0038747	0.2935	36	0.009	2.046	*ZNF608*	BMI/obesity
10	40,710,979	40,868,229	MARC0038064	ALGA0058443	0.0985	149	0.009	2.046	*LINGO2*	BMI/obesity
15	142,893,866	142,992,475	MARC0112236	ALGA0087841	0.3401	29	0.009	2.046	*KPNA4*	
2	157,110,989	157,223,320	MARC0039166	M1GA0003373	0.2571	40	0.01	2.000	*ABLIM3*	
2	157,270,386	157,362,402	H3GA0008275	ALGA0017005	0.4738	17	0.01	2.000	*AFAP1L1*	obesity

^1^Start and End refer to SNP position in Sus scrofa Build 10.2

^2^Windows are ranked is by % genetic variance

Twenty-eight of the QTL detected were within 1 Mb of other significant QTL (ranging from two to six 5-SNP windows within 1 Mb of a significant QTL). These adjacent groups of QTL may actually be due to a single quantitative trait nucleotide or gene in the region. High LD between markers in adjacent 5-SNP windows was observed in six QTL regions on SSC2 from 97.3–99.7 Mb, SSC6 from 88–89 Mb, SSC8 from 71.8–73.8 Mb, SSC9 from 93–96 Mb, SSC10 from 35.6–37.7 Mb and on SSC16 from 26–27.3 Mb (Additional file 3: Figure S2). The region on SSC2 at 98 Mb was previously associated with delayed puberty in pigs [27].

Sixteen of the QTL were within 0.5 Mb of previously reported associations [27] from the same population where attainment of puberty was considered a categorical trait and a case–control experimental design was implemented. Animals from the previous study that had not reached puberty were not included in the current study as their age at puberty was unknown. Two associations were located in published QTL for age of puberty in Meishan x European pig resource populations on SSC1 at 292 Mb [39] and on SSC10 at 68.5-69.5 Mb [40, 41]. Eight regions corresponded to those previously reported by Tart et al. [26] in a genome-wide association study (Additional file 2: Table S1).

### Candidate genes in QTL regions

The most significant QTL accounting for 9.7 % of the genetic variance was located on SSC12 at 15 Mb within the growth hormone (*GH1*) and chorionic somatomammotropin hormone (*CSH1*) gene cluster. Growth hormone and its receptor are necessary for the onset and normal course for attainment of puberty [42]. There are age related changes in somatotropin secretion in pigs, with serum concentrations of GH declining as gilts approach pubertal age [43–45]. A second QTL accounting for over 7.1 % of the genetic variance was located on SSC7 at 75 Mb near the *PRKD1* and *C14orf 23* loci. *PRKD1* is associated with body-mass index (BMI) in humans [46] and an SNP in this window (INRA0026430) near *C14orf23* was previously reported to be associated with delayed puberty in relatives of the same population of pigs [27].

For regions previously reported by Tart et al. [26] likely candidate genes are *CRTC1* (SSC2:59 Mb), *PAPPA* (SSC1:288 Mb), *CCT6A* (SSC3:17 Mb), *CELF4* (SSC6:115 Mb), *EDEM3* (SSC9:139 Mb), *SMYD2* (SSC9:142 Mb), and *XXYLT1* (SSC13:142 Mb). *CCT6A* expression was shown to be higher in sexually mature laying hen ovaries than in immature ovaries [47].

Six regions contained genes associated with age at menarche in humans, and identified by Tart et al. [26] as well as the current study to be associated with age at puberty in pigs. *CRTC1* (SSC2:58.9 Mb) was associated with age at menarche in humans by Elks et al. [22]. This gene also leads to infertility (failure to ovulate) in mice that lack a functional *CRTC1* [48] through mediation of the leptin-kisspeptin-GnRH pathway. Kisspeptin is a key regulator of LH secretion in the gilt [49]. *IQCH* located on SSC 1 at 183 Mb was associated with age at menarche in humans [22, 23]. In addition, three SNP in *IQCH* form a haplotype associated with a major gene affecting ovulation rate in twinner cattle [50]. *RORA* at 122 Mb on SSC1 was associated with age at menarche [22, 23] while a deletion in *RORA* in staggerer mice causes delayed puberty, reduced oocyte number and accelerated reproductive aging [51]. Finally, *AQP8* at 22 Mb on SSC3 [22] and *GPRC5B* at 27 Mb on SSC3 [24] have been associated with age at menarche while *NEGR1* at 130 Mb on SSC6 was associated with age at menarche and BMI [22, 46].

Several loci that are associated with anthropometric traits in humans such as body-mass index (BMI) or its components, body mass (obesity) and height were also associated with pubertal age in pigs (Additional file 2: Table S1). These include *MTCH2* and *ZNF608* on SSC2 at 16 Mb (BMI and obesity) [46, 52], and 133 Mb (BMI) [46, 52], *NEGR1* on SSC6 at 130 MB (obesity) [53], *ADAMTSL3* and *PRKD1* on SSC7 at 57 Mb (BMI and height) [54, 55] and 75 Mb [46], and *NPFFR2* on SSC8 at 72 Mb (BMI) [56]. Several human studies have shown relationships between the onset of puberty and adolescent growth rate [57], BMI [58] and obesity [59]. Using Mendelian randomization, Mumby et al. [60] showed a causative effect of increased BMI on earlier age at menarche in humans. Some studies in pigs have shown that gilts with higher growth rates [61, 62] or fed high energy diets [63] reach puberty earlier than lower growth rate or food restricted gilts [16]. Although faster growing gilts typically reach puberty earlier, there is little experimental evidence for a discrete level of body fatness necessary for puberty to occur in commercial pigs when lean tissue growth is not limiting [61, 64]. Metabolic state at critical periods of development [65,66] or the degree of positive energy balance as determined by lifetime growth rate [62, 67] are considered more important determinants of pubertal age in gilts. Many loci associated with human age at menarche, BMI or obesity are involved in energy balance, such as *CRTC1* [48], *NEGR1* [53], *MTCH2* [68], *RORA* [69] and *TBC1D1* [70]. Variation in *TBC1D1* and *ZNF608* has been shown to be associated with fatness traits in pigs [71, 72]. An SNP window about 200 kb proximal to leptin (LEP) on SSC18 at 20.9 Mb was associated with age at puberty. Leptin levels and age at puberty in the pig are genetically correlated [20] and an increase in leptin seems to be permissive for pubertal development in the pig [73]. When lines of pigs were experimentally selected for increased lean percentage and less backfat thickness, gilts were older at puberty [74] owing to a general negative effect on the intensity of expressed estrous behavior [75, 76]. Reduced intensity of expressed estrous behavior would make it harder to detect pubertal estrus. Given that expression of estrus behavior was used to phenotype age at puberty in the current study, identification of loci linking age at puberty with body fatness or metabolism may not be directly due to activation of the hypothalamic-pituitary-gonadal axis per se, but rather a result of associated effects on sexual behavior.

Several other candidate genes were identified that are involved in pubertal development in other species. Two locations coincided with genes associated with puberty in cattle; *IGF1R* on SSC1 at 153 Mb [77] and *ESRRG* on SSC10 at 9 Mb [78]. In rats, hypothalamic blockade of *NELL2* (on SSC5 at 78.5 Mb) expression reduces GnRH release and results in delayed puberty [79, 80]. Alpha-fetoprotein (*AFP*) on SSC8 at 73.7 Mb is essential for female fertility [81] as mutant female mice fail to ovulate. Markers in this region on SSC8 showed high linkage disequilibrium that extended for about 2 Mb (71.8–73.8 Mb) and includes the genes *NPFFR2*, *ADAMTS3*, *ALB* and *AFP*. Substance P encoded by the tachykinin gene (*TAC1*) on SSC9 at 85 Mb and activin receptor-like kinase 7 (*ACVR1C*) on SSC15 at 71 Mb are associated with delayed puberty in female knockout mice [82, 83]. Substance P in found in the preoptic and suprachiasmatic areas in the medial forebrain of the pig [84], which are areas of the porcine hypothalamus that contain GnRH neurons [85]. Substance P is also found in the adenohypophysis of the pig [86] where it acts directly on porcine gonadotrope cells to modulate secretion of LH [87]. Moreover, substance P reportedly attenuated growth-hormone releasing hormone stimulated secretion of GH in primary cultures of porcine pituitary cells [88]. Given that puberty in the gilt is accompanied with a reduction in GH secretion and increased LH secretion [89], these data are strong supportive evidence for the association of substance P with age at puberty in the pig found in the current study.

## Conclusions

Although rare monogenic mutations can disrupt normal pubertal development in humans through the GnRH axis, most loci contain common genetic variants that contribute to variation in pubertal timing and are involved in co-regulation of height, BMI or obesity (reviewed in Day et al.) [90]. Results of the current study illustrate the complex relationship of growth and metabolism with puberty in the pig. Several of the loci identified to be associated with age at puberty in gilts in this study contain candidate genes that can have direct actions at the anterior pituitary gland or interact centrally with the kisspeptin-GnRH neural network to control secretion of LH and many loci were identified that have a physiological role for the onset of puberty in rodents, cattle and pigs and a genetic basis for sexual maturation in humans. This provides strong correlative evidence that these genomic associations in the pig are reliable. The genomic markers identified in the current experiment are an important resource that will be used to develop validated markers for age at puberty in commercial populations of pigs. Because age at puberty is not routinely measured or selected for in commercial herds, and is a predictive factor for sow longevity [41, 91] and lifetime productivity [16], these genomic markers will facilitate the use of genomic or marker-assisted selection to improve these traits.

### Availability of data and materials

All relevant data are available within the manuscript and its Supporting Information files.

## Additional files

Additional file 1: Figure S1. Genotypic principal components analysis of 759 phenotyped animals; the first three principal components are plotted. (PDF 197 kb) Additional file 2: Table S1. Genomic regions, SNP results and candidate genes associated with age at puberty in pigs. (XLSX 37 kb) Additional file 3: Figure S2. Linkage disequilibrium plots of QTL regions of more than one consecutive 5-SNP window greater that 1 Mb with high r^2^ values. (PDF 821 kb)

## Abbreviations

ARS: Agricultural Research Service
BARC: Beltsville Agricultural Research Center
BMI: body-mass index
GV: genetic variance
LD: linkage disequilibrium
SSC: Sus scrofa chromosome
USDA: United States Department of Agriculture
USMARC: U.S. Meat Animal Research Center
