# Supplementary figures and images for: Genome-wide association and identification of candidate genes for age at puberty in swine
Source: BMC Genet. 2016 Feb 29;17:50. doi: 10.1186/s12863-016-0352-y (PMC4770536; doi:10.1186/s12863-016-0352-y)

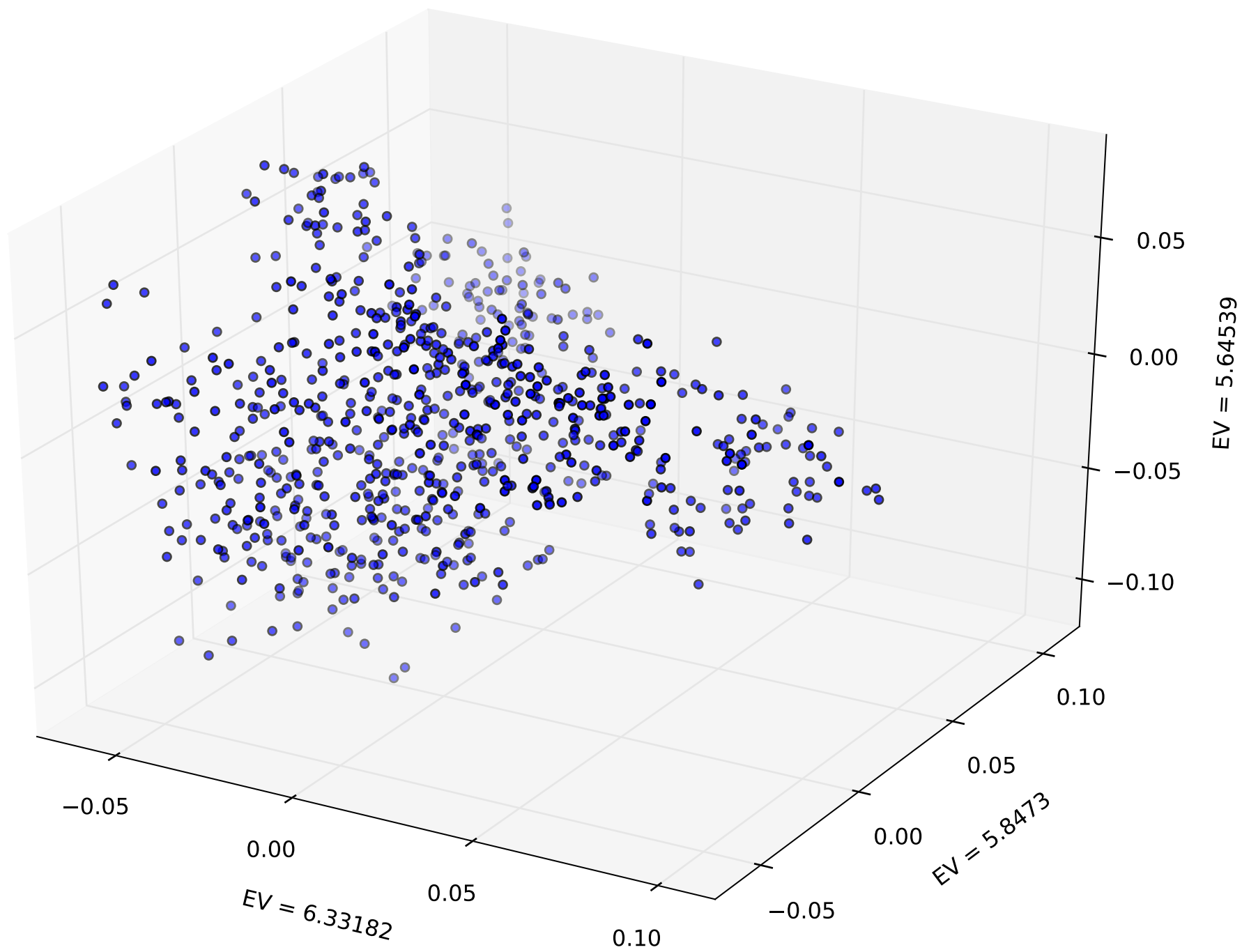

Supplement: Additional file 1: Figure S1. — Genotypic principal components analysis of 759 phenotyped animals; the first three principal components are plotted. (PDF 197 kb) [file 12863_2016_352_MOESM1_ESM.pdf]

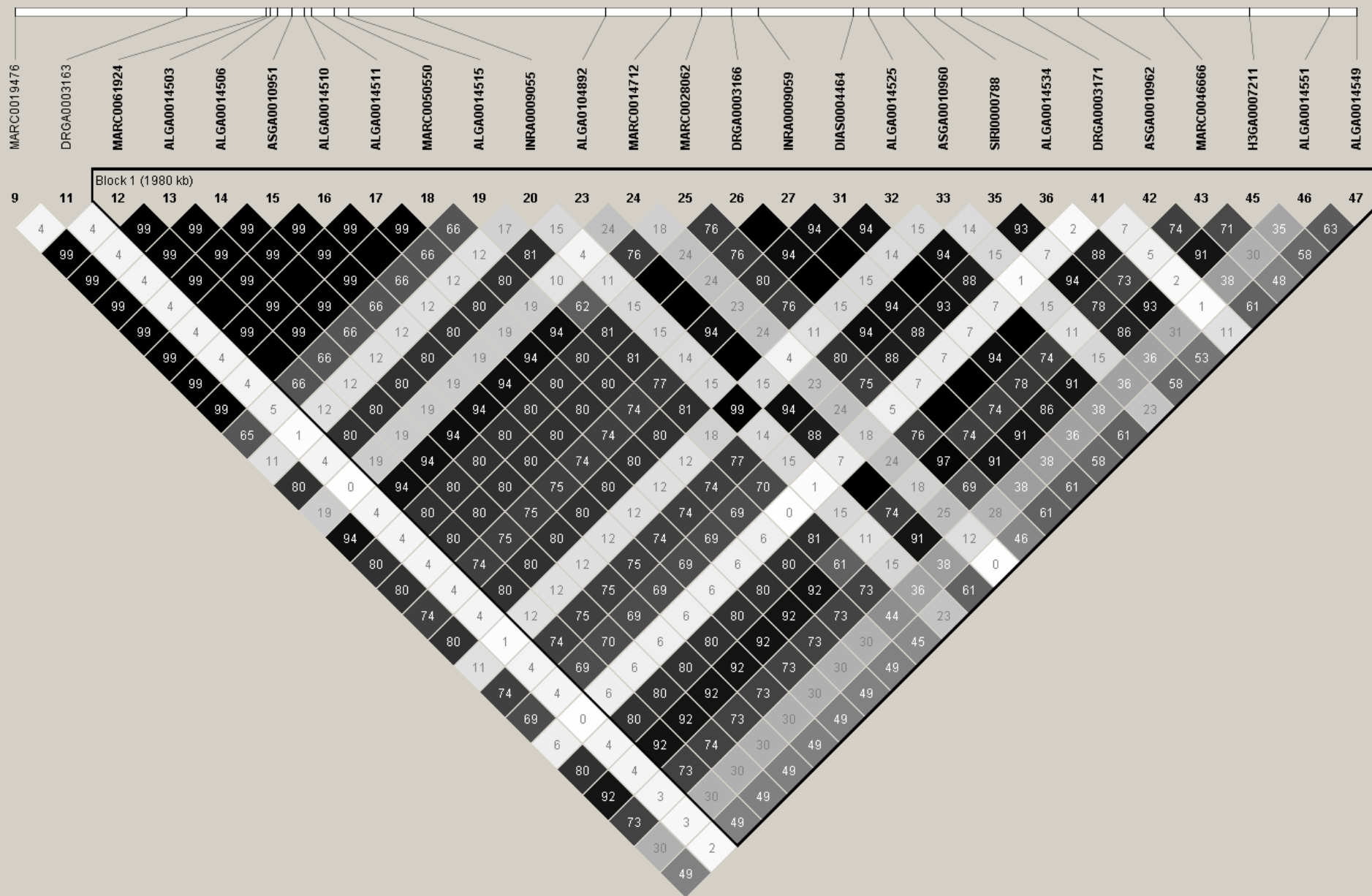

SSC2\_97-99

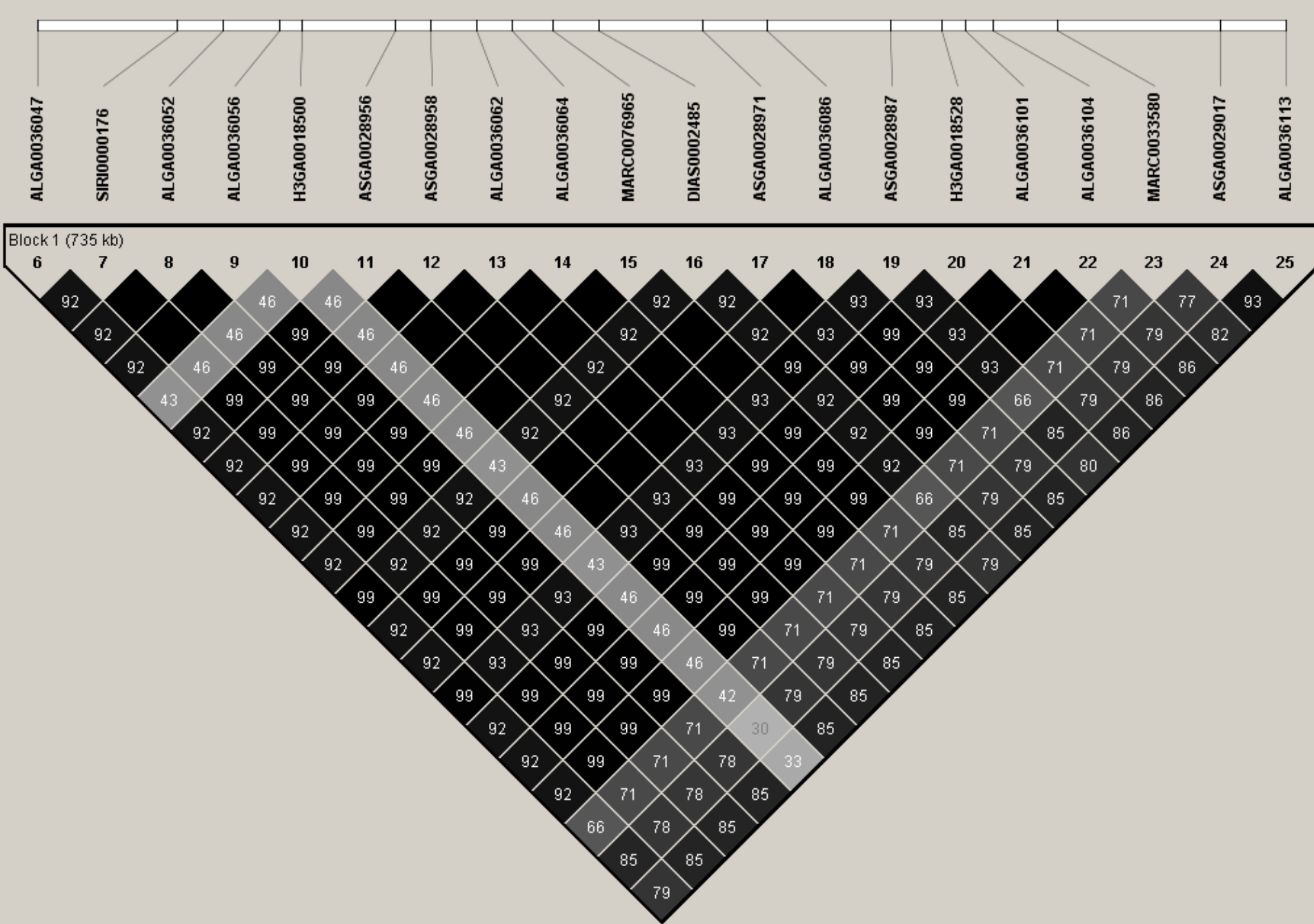

SSC6\_88-89

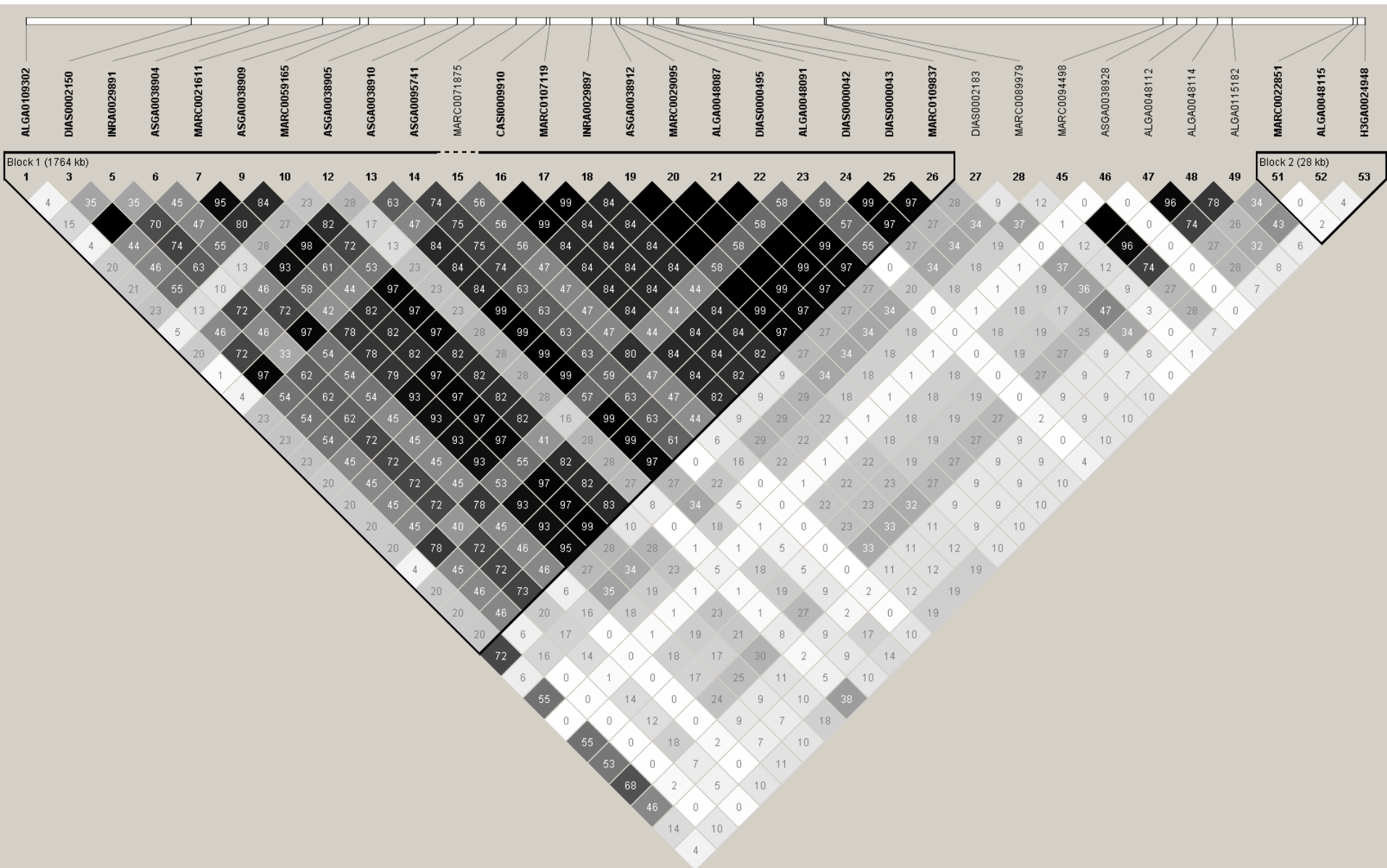

SSC8\_71.8-73.8

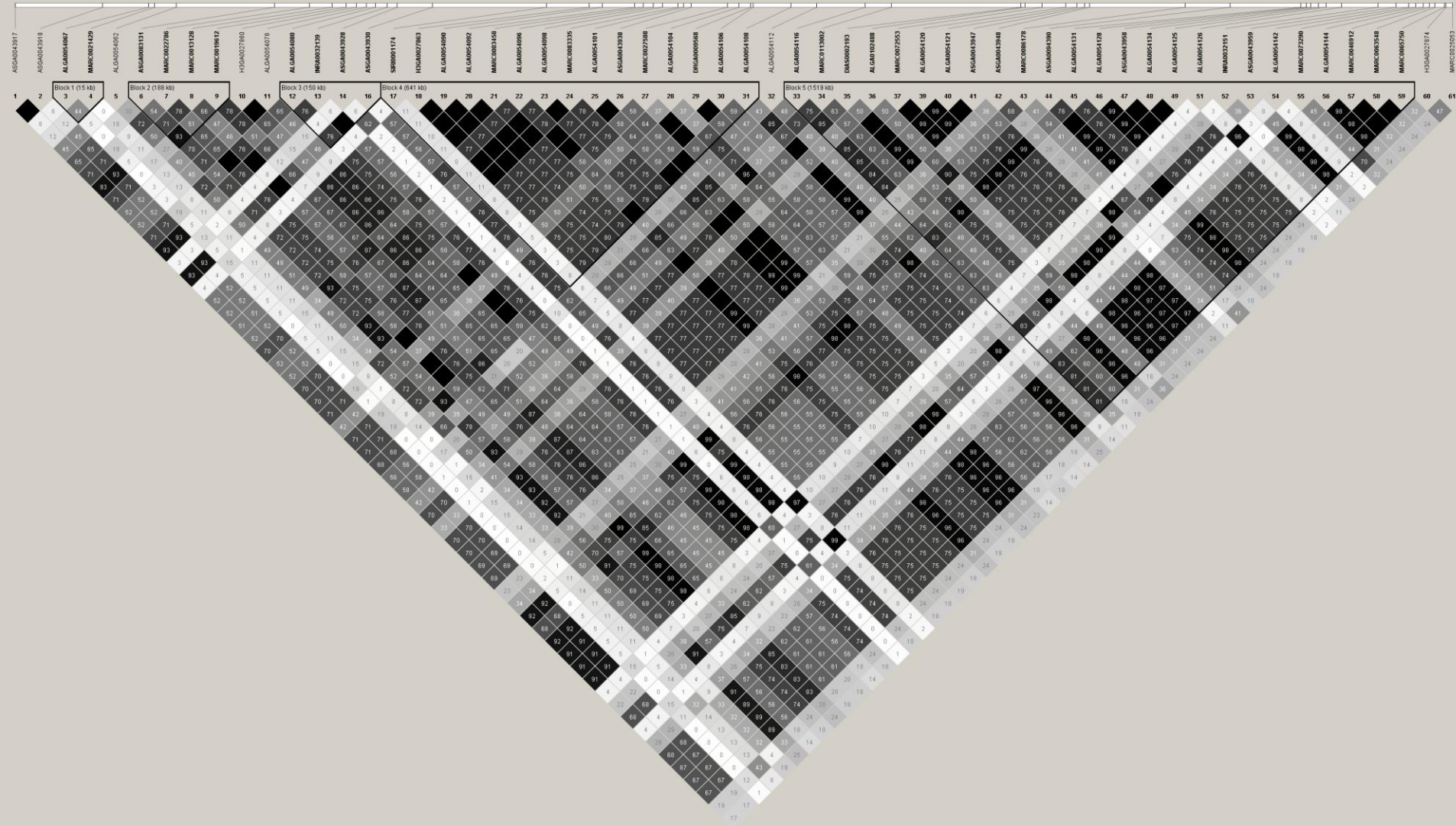

SSC9\_92-96

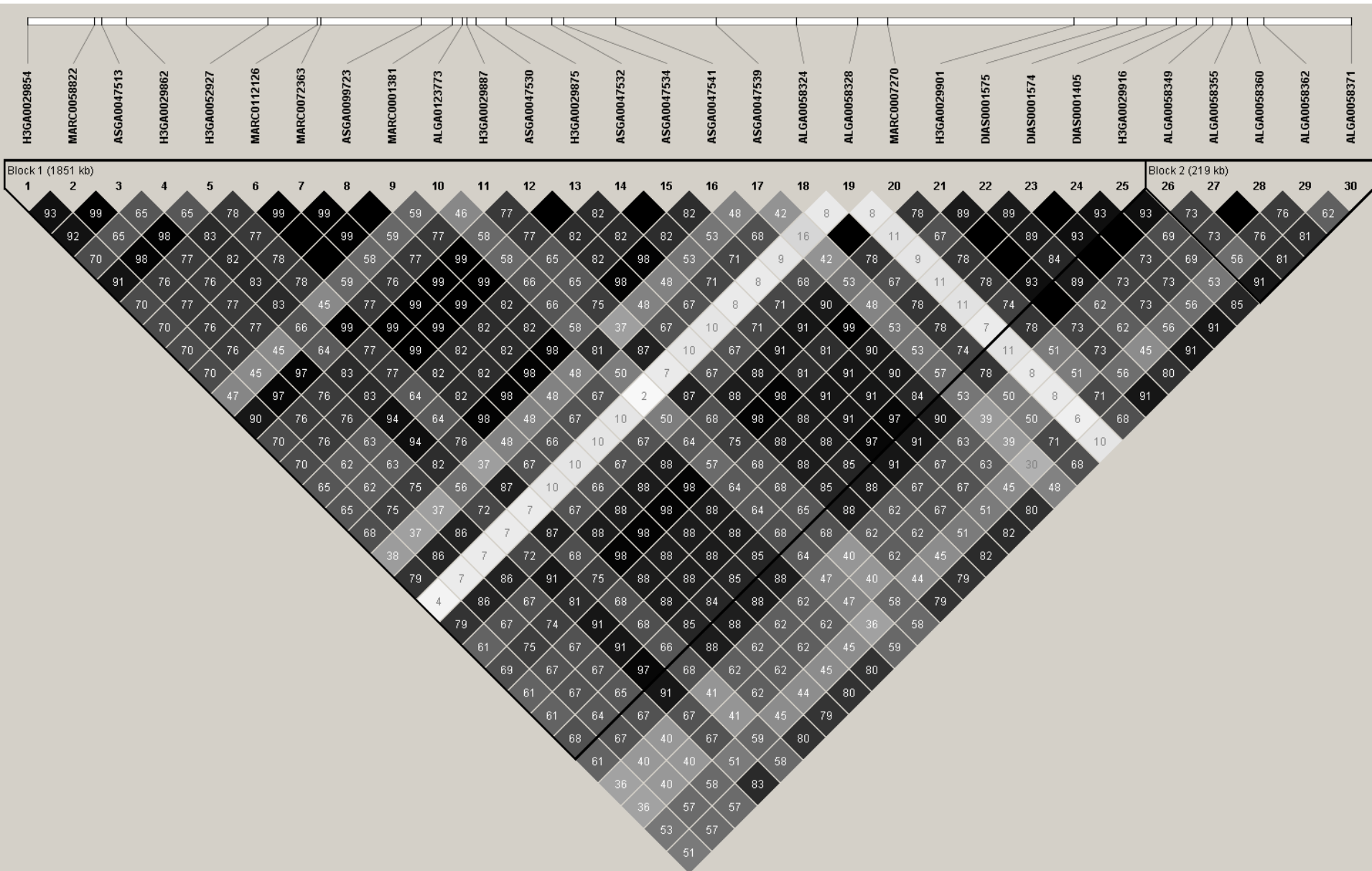

SSC10\_35-37

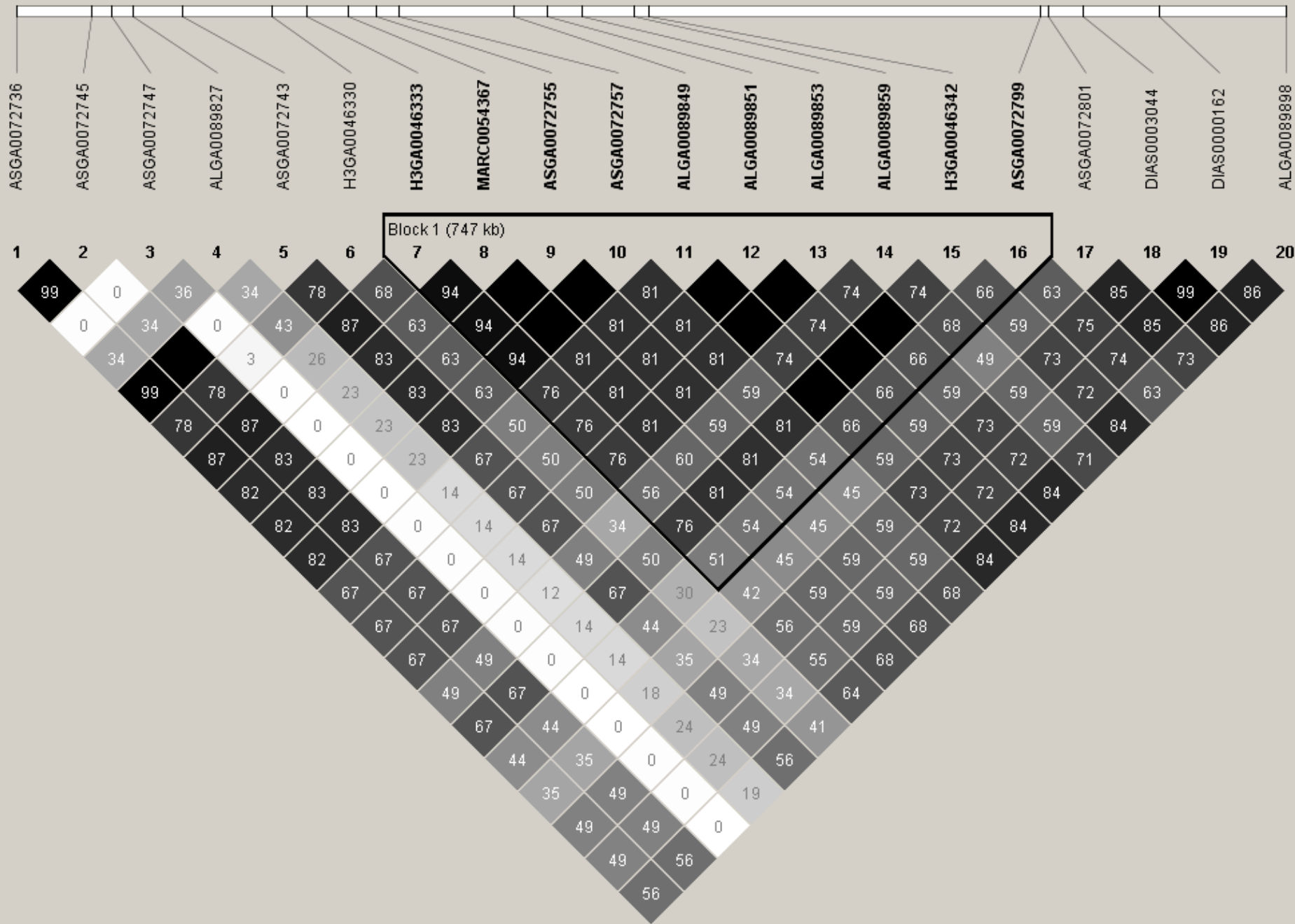

SSC16\_26-27

Supplement: Additional file 3: Figure S2. — Linkage disequilibrium plots of QTL regions of more than one consecutive 5-SNP window greater that 1 Mb with high r2 values. (PDF 821 kb) [file 12863_2016_352_MOESM3_ESM.pdf]
